# Chemical Composition and Insecticidal Activity Against *Sitophilus zeamais* of the Essential Oils Derived from *Artemisia giraldii* and *Artemisia subdigitata*

**DOI:** 10.3390/molecules17067255

**Published:** 2012-06-13

**Authors:** Sha-Sha Chu, Zhi-Long Liu, Shu-Shan Du, Zhi-Wei Deng

**Affiliations:** 1 Department of Entomology, China Agricultural University, Haidian District, Beijing 100193, China; 2 State Key Laboratory of Earth Surface Processes and Resource Ecology, Beijing Normal University, Beijing 100875, China; 3 Analytical and Testing Center, Beijing Normal University, Beijing 100875, China

**Keywords:** *Artemisia giraldii*, *Artemisia subdigitata*, *Sitophilus zeamais*, fumigant, contact toxicity, essential oil composition

## Abstract

The aim of this research was to determine the chemical composition and insecticidal activity of the essential oils derived from flowering aerial parts of *Artemisia giraldii* Pamp. and *A. subdigitata* Mattf. (Family: Asteraceae) against the maize weevil (*Sitophilus zeamais* Motsch.). Essential oils of aerial parts of *A. giraldii* and *A. subdigitata* were obtained from hydrodistillation and investigated by GC and GC-MS. A total of 48 and 33 components of the essential oils of *A. giraldii* and *A. subdigitata* were identified, respectively. The principal compounds in *A. giraldii* essential oil were β-pinene (13.18%), *iso*-elemicin (10.08%), germacrene D (5.68%), 4-terpineol (5.43%) and (*Z*)-β-ocimene (5.06%). 1,8-Cineole (12.26%) and α-curcumene (10.77%) were the two main components of the essential oil of *A. subdigitata*, followed by β-pinene (7.38%), borneol (6.23%) and eugenol (5.87%). The essential oils of *A. giraldii* and *A. subdigitata* possessed fumigant toxicity against the maize weevils with LC_50_ values of 6.29 and 17.01 mg/L air, respectively. The two essential oils of *A. giraldii* and *A. subdigitata* also exhibited contact toxicity against *S. zeamais* adults with LD_50_ values of 40.51 and 76.34 µg/adult, respectively. The results indicated that the two essential oils show potential in terms of fumigant and contact toxicity against grain storage insects.

## 1. Introduction

The maize weevil (*Sitophilus zeamais* Motsch.) is a serious pest of stored grains worldwide. Infestations not only cause significant economic losses due to the consumption of grains; they also result in elevated temperature and moisture conditions that lead to an accelerated growth of molds, including toxigenic species [1]. The maize weevils are small (2.5 mm to 4 mm length) brown black weevils with a long slender snout and four reddish brown spots on the wing covers (two spots on each wing cover). The head and thorax are nearly as long as the wing covers. They are a primary pest of grain as they can infest undamaged grain. In China, they are a major pest of corn. Control of the stored product insects now is based on the application of synthetic insecticides/fumigants. However, repeated use of those fumigants/insecticides for decades has led to resurgence of stored-product insect pests, sometimes resulted in the development of resistance, and had undesirable effects on non-target organisms [2]. These problems have highlighted the need to develop new types of selective insect-control alternatives with fumigant action. Plant essential oils and their components have been shown to possess potential to be developed as new fumigants and they may have the advantage over conventional fumigants in terms of low mammalian toxicity, rapid degradation and local availability [3]. More than 15 essential oils derived from plant species of the genus Artemisia have been evaluated for insecticidal activities against stored product insects [4,5,6,7,8,9,10,11,12,13,14].

The genus *Artemisia* (commonly wormwood or sagebrush) is one of the largest and most widely distributed genera of the family Asteraceae. It comprises about 380 species of herbs and shrubs well-known for their volatile oil that is extensively used in food and pharmaceutical industry. *Artemisia giraldii* Pamp. and *A. subdigitata* Mattf. are two of the 186 species of *Artemisia* found in China [15]. *A. giraldii* is an herbaceous plant distributed only in some areas of China (e.g., Henan, Hebei, Gansu, Ningxia, Shannxi, and Sichuan province). In the previous studies, two flavones and several monoterpenoids and sesquiterpenoids were isolated from *A. giraldii* aerial parts and identified [16,17,18,19]. *A. subdigitata* is a species of perennial herbaceous rhizome plant distributed in some areas of China (e.g., Gansu, Guizhou, Hebei, inner Mongol, Sichuan, and Yunnan province) and also Nepal, Bhutan, and North India. It is used as traditional medicinal herb in some areas of China (Folium Artemisiae Argyi) [15]. It is used in Chinese traditional medicine to stop bleeding by warming meridians, expel cold and alleviate pain, and prevention of miscarriages [15]. Most of the components isolated from the extracts of *A. subdigitata* were terpenoids [20,21,22,23,24].

During our mass screening program for new agrochemicals from the wild plants, essential oils of *A. giraldii* and *A. subdigitata* were found to possess strong insecticidal activity against maize weevils. A literature survey showed that there are no reports on the volatile constituents and insecticidal activity of *A. giraldii*. The chemical composition of the essential oil derived from *A. subdigitata* has been previously reported [25,26]. However, to date, there has been no report on the insecticidal activity of *A. subdigitata* essential oil. Thus we decided to investigate the chemical constituents and insecticidal activity of the essential oils of *A. giraldii* and *A. subdigitata* against grain storage insects for the first time.

## 2. Results and Discussion

The yellow essential oil yield of *A. giraldii* flowering aerial parts was 0.36% v/w and the density of the concentrated essential oil was determined to be 0.85 g/mL. The essential oil yield of *A. subdigitata* was 0.64% v/w and the density of the oil was 0.79 g/mL. A total of 48 components of the essential oil of *A. giraldii* flowering aerial parts were identified (Table 1). The principal compounds in *A. giraldii* essential oil were β-pinene (13.18%), *iso*-elemicin (10.08%), germacrene D (5.68%), 4-terpineol (5.43%) and (*Z*)-β-ocimene (5.06%). Monoterpenoids represented 24 of the 48 compounds, corresponding to 53.57% of the whole oil, while 21 of the 48 constituents were sesquiterpenoids (41.49% of the crude essential oil). The main constituents in *A. giraldii* essential oil were quite different from those in the essential oils derived from the other species of the genus *Artemisia* from China. For example, the main components of essential oil of *A. vestita* were grandisol (40.3%), 1,8-cineol (14.9%) and camphor (11.4%) [10]. In another report, the major components of *A. lavandulaefolia* oil were caryophyllene (15.5%), β-thujone (13.8%), 1,8-cineole (13.1%), and β-farnesene (12.3%), and the principal compounds identified in *A. sieversiana* oil were 1,8-cineole (9.2%), geranyl butyrate (9.2%), borneol (7.9%), and camphor (7.9%) [13]. However, the main components of the essential oil of *A. eriopoda* were germacrene D (21.6%) and 1,8-cineole (14.2%) [14] while the principal compounds in the essential oil of *A. igniaria* were 1,8-cineole (14.4%) and camphor (13.4%) [27]. 

A total of 33 components of the essential oil of *A. subdigitata* were identified, accounting for 98.31% of the total oil (Table 2). 1,8-Cineole (12.26%) and α-curcumene (10.77%) were the two main constituents of *A. subdigitata* essential oil, followed by β-pinene (7.38%), borneol (6.23%) and eugenol (5.87%). The chemical composition of the essential oil of *A. subdigitata* was quite different from that reported in other studies. For example, β-pinene (35.7%) and limonene (11.0%) was the two main components of the essential oils of young leaves of *A. subdigitata* harvested in Northwest China [20]. However, the leaf oil of *A. subdigitata* collected from Mongolia was dominated by eugenol (11.2%), methyl eugenol (9.4%) and camphor (9.0%) [21]. The above findings suggested that there were great geographic variations in chemical composition of *A. subdigitata* essential oil.

The essential oils of *A. giraldii* and *A. subdigitata* flowering aerial parts exhibited contact toxicity against *S. zeamais* adults, with LD_50_ values of 40.51 and 76.34 μg/adult, respectively (Table 3). The essential oil of *A. giraldii* shows stronger toxicity than *A. subdigitata* essential oil against *S. zeamais* adults (no overlaps in 95% fiducial limit, Table 3). Compared with the famous botanical insecticide, pyrethrum extract (25% pyrethrine I and pyrethrine II), the two essential oils were 9 and 18 times less toxic against the maize weevils because pyrethrum extract displayed a LD_50_ value of 4.29 μg/adult [12]. 

The essential oils of *A. giraldii* and *A. subdigitata* flowering aerial parts also possessed strong fumigant activity against *S. zeamais* adults, with LC_50_ values of 6.29 and 17.01 mg/L air, respectively (Table 3). On the basis of LC_50_ values, *S. zeamais* adults were significantly more susceptible (no overlaps in 95% fiducial limit) to the essential oil of *A. giraldii* than to *A. subdigitata* essential oil. However, the currently used grain fumigant, methyl bromide (MeBr) was reported to have fumigant activity against *S. zeamais* adults with a LC_50_ value of 0.67 mg/L air [28]. Thus, the two essential oils of *A. giraldii* and *A. subdigitata* flowering aerial parts were only 9 and 25 times less toxic to the maize weevil compared with the commercial fumigant MeBr. However, compared with the other essential oils in the previous studies, the two essential oils of *A. giraldii* and *A. subdigitata* exhibited stronger or the same level of fumigant toxicity against the maize weevils, e.g., essential oils of *Murraya exotica* (LC_50_ = 8.29 mg/L) [29], *A. lavandulaefolia* LC_50_ = 11.2 mg/L) and *A. sieversiana* (LC_50_ = 15.0 mg/L) [13], *A. vestita* (LC_50_ = 13.42 mg/L) [10], *A. capillaris* (LC_50_ = 5.31 mg/L) and *A. mongolica* (LC_50_ = 7.35 mg/L) [12], *Illicium simonsii* (LC_50_ = 14.95 mg/L) [11], *I. fragesii* (LC_50_ = 11.36 mg/L) [30], *Rhododendron anthopogonoides* (LC_50_ = 9.66 mg/L) [31], *Kadsura heteroclite* (LC_50_ = 14.04 mg/L) [32], and *Lonicera japonica* (LC_50_ = 13.36 mg/L) [33]. The above findings suggest that fumigant activity of the two essential oils is quite promising, considering the currently used fumigants are synthetic insecticides and they show potential to be developed as possible natural fumigants for the control of stored product insects. Moreover, for the practical application of the essential oils as novel fumigant/insecticide, further studies on the safety of the essential oils to humans and on development of formulations are necessary to improve the efficacy and stability and to reduce cost. The isolation and identification of the bioactive compounds in the essential oils of *A. giraldii* and *A. subdigitata* flowering aerial parts are of utmost importance so that their potential application in controlling stored-product pests can be fully exploited.

In previous studies [6,34,35,36,37,38,39,40], the insecticidal activity of 1,8-cineole, β-pinene, borneol, terpinen-4-ol, and (*Z*)-β-ocimene (main constituents of the studied essential oils) against the grain storage insects had been reported. For example, 1,8-cineole and β-pinene exhibited fumigant toxicity against *S. zeamais* adults, with 24 h LC_50_ values of 1.82 mg/cm^2^ and 3.82 mg/cm^2^, respectively [34] and also possessed contact toxicity against *S. zeamais* adults with 7 d LD_50_ values of 48 μg/mg and 113 μg/mg, respectively, while terpinen-4-ol has a 7 d LD_50_ value of 10 μg/mg [35]. Moreover, (*Z*)-β-ocimene exhibited fumigant toxicity against *S. zeamais* adults with a 24 h LC_50_ value of 28.66 mg/L air [36]. However, no reports on insecticidal activity of α-curcumene, *iso*-elemicin and germacrene D against grain storage insects were available so far. 

## 3. Experimental

### 3.1. Plant Material and Essential Oil Extraction

Fresh flowering aerial parts (10 kg of leaves, stems and flowers) of *A. giraldii* and *A. subdigitata* were harvested in August 2010 from Xiaolongmeng National Forest Park (Mentougou District, Beijing 102300). The samples were air-dried for one week and ground to a powder using a grinding mill (Retsch Muhle, Haan, Germany). The species were identified by Dr Liu, QR (College of Life Sciences, Beijing Normal University) and the voucher specimens (BNU-zhilongliu-2010-08-23-035 and BNU-zhilongliu-2010-08-23-036) were deposited at the Herbarium (BNU) of College of Life Sciences, Beijing Normal University. The ground powder of the two plants was subjected to hydrodistillation using a modified Clevenger-type apparatus for 4 h and extracted with *n*-hexane. Anhydrous sodium sulphate was used to remove water after extraction. Essential oils were stored in airtight containers in a refrigerator at 4 °C. 

### 3.2. Insects

The maize weevils (*S. zeamais*) were obtained from laboratory cultures maintained for the last 15 years in the dark in incubators at 29–30 °C and 70–80% relative humidity. The maize weevils were reared on whole wheat at 12–13% moisture content in glass jars (diameter 85 mm, height 130 mm) at 29–30 °C and 70–80% relative humidity. Unsexed adult weevils used in all the experiments were about 2 weeks old.

### 3.3. Gas Chromatography-Mass Spectrometry

The two essential oils were subjected to GC-MS analysis on an Agilent system consisting of a model 6890N gas chromatograph, a model 5973N mass selective detector (EIMS, electron energy, 70 eV), and an Agilent ChemStation data system. The GC column was an HP-5ms fused silica capillary with a 5% phenyl-methylpolysiloxane stationary phase, film thickness of 0.25 µm, a length of 30 m, and an internal diameter of 0.25 mm. The GC settings were as follows: The initial oven temperature was held at 60 °C for 1 min and ramped at 10 °C·min^−1^ to 180 °C held for 1 min, and then ramped at 20 °C·min^−1^ to 280 °C and held for 15 min. The injector temperature was maintained at 270 °C. The sample (1 μL) was injected neat, with a split ratio of 1:10. The carrier gas was helium at flow rate of 1.0 mL·min^−1^. Spectra were scanned from 20 to 550 m/z at 2 scans·s^−1^. Most constituents were identified by gas chromatography by comparison of their retention indices with those of the literature or with those of authentic compounds available in our laboratories. The retention indices were determined in relation to a homologous series of *n*-alkanes (C_8_–C_24_) under the same operating conditions. Further identification was made by comparison of their mass spectra with those stored in NIST 05 and Wiley 275 libraries or with mass spectra from literature [41]. Component relative percentages were calculated based on normalization method without using correction factors.

### 3.4. Contact Toxicity

The contact toxicity of the two essential oils against *S. zeamais* adults was measured as described by Liu and Ho [28]. Range-finding studies were run to determine the appropriate testing concentrations. A serial dilution of the essential oil (six concentrations, 2.6–13.3%, v/w) was prepared in *n*-hexane. Aliquots of 0.5 μL of the dilutions were applied topically to the dorsal thorax of the insects, using a Burkard Arnold microapplicator. Controls were determined using 0.5 µL of *n*-hexane per insect. Ten insects were used for each concentration and control, and the experiment was replicated six times. Both treated and control insects were then transferred to glass vials (10 insects/vial) with culture media and kept in incubators at 29–30 °C and 70–80% relative humidity. Mortality was observed after 24 h. The observed mortality data were corrected for control mortality using Abbott’s formula. Results from all replicates were subjected to probit analysis using the PriProbit Program V1.6.3 to determine LD_50_ values [42].

### 3.5. Fumigant Toxicity

Range-finding studies were run to determine the appropriate testing concentrations. A serial dilution of the essential oil (2.0–40.0%, six concentrations) was prepared in *n*-hexane. A Whatman filter paper (diameter 2.0 cm) was placed on the underside of the screw cap of a glass vial (diameter 2.5 cm, height 5.5 cm, volume 24 mL). Ten microliters of an appropriate concentration of the essential oil was added to the filter paper. The solvent was allowed to evaporate for 15 s before the cap was placed tightly on the glass vial (with 10 insects) to form a sealed chamber. Fluon (ICI America Inc., Wilmington, DE, USA) was used inside each glass vial to prevent insects from the treated filter paper. Preliminary experiments demonstrated that 15 s were sufficient for the evaporation of solvents. *n*-Hexane was used as controls. Six replicates were used in all treatments and controls and they were incubated at 29–30 °C and 70–80% relative humidity for 24 h. The mortality was recorded. The observed mortality data were corrected for control mortality using Abbott’s formula. Results from all replicates were subjected to probit analysis using the PriProbit Program V1.6.3 to determine LC_50_ values [42].

## 4. Conclusions

The composition of the essential oils obtained from *A. giraldii* and *A. subdigitata* flowering aerial parts was determined by GC-FID and GC-MS. The two essential oils exhibited strong fumigant activity against *S. zeamais* adults. The two essential oils of *A. giraldii* and *A. subdigitata* were only 9 and 25 times less toxic to the maize weevil compared with the commercial fumigant MeBr. Moreover, the essential oils exhibited stronger toxicity than most of the reported essential oils in the literature. The two essential oils also possessed strong contact toxicity against the maize weevils. These findings suggest that the essential oils of *A. giraldii* and *A. subdigitata* flowering aerial parts have potential for development as novel natural insecticides/fumigants for grain storage insects. Further studies are required to isolate and identify the active components from the essential oils.

## Figures and Tables

**Table 1:** Constituents identified from the essential oil of *Artemisia giraldii* aerial parts.

RI *	Compound	Percent Composition
939	α-Pinene	3.52
981	β-Pinene	13.18
1028	3-Isopropenyl-5,5-dimethylcyclopentene	3.01
1038	(*Z*)-β-Ocimene	5.06
1044	β-Terpinene	0.15
1057	γ-Terpinene	1.21
1067	*cis*-Linalool oxide	1.64
1088	Terpinolene	0.80
1094	Linalool	3.36
1112	Rose oxide	3.72
1117	2,6-Dimethyleneoct-7-en-3-one	0.22
1140	*trans*-p-Menth-2-en-1-ol	0.35
1147	*allo*-Ocimene	0.72
1167	Borneol	0.28
1175	4-Terpineol	3.87
1182	*p*-Cymen-8-ol	5.43
1191	α-Terpineol	0.95
1226	*cis*-Geraniol	1.66
1275	Verbenyl acetate	1.82
1281	Phellandral	0.13
1289	Bornyl acetate	0.2
1326	Methyl geranate	0.18
1362	3-Allylguaiacol	0.96
1365	Neryl acetate	0.67
1383	*trans*-Geranyl acetate	1.24
1388	β-Cubebene	0.36
1392	*cis*-Jasmone	0.35
1420	Caryophyllene	3.38
1437	α-Guaiene	1.31
1440	*epi*-Bicyclosesquiphellandrene	0.81
1452	*trans*-β-Farnesene	2.06
1454	1,4,7,-Cycloundecatriene, 1,5,9,9-tetramethyl-, *Z*,*Z*,*Z*-	0.77
1468	γ-Himachalene	1.08
1474	*ar*-Curcumene	1.81
1480	Germacrene D	5.68
1498	α-Muurolene	0.21
1500	Methyl isoeugenol	2.19
1505	α-Farnesene	2.83
1513	γ-Cadinene	0.55
1524	β-Sesquiphellandrene	2.63
1537	*trans*-Cadina-1,4-diene	0.43
1554	Elemicin	0.55
1563	Nerodilol	1.03
1578	Spathulenol	2.05
1596	*iso*-Elemicin	10.08
1623	3,4,5-trimethoxy-Benzaldehyde	0.46
1653	α-Cadinol	2.38
1714	Eudesma-4,11-dien-2-ol	1.59
	Total identified	98.92
	Monoterpenoids	53.57
	Sesquiterpenoids	41.49
	Others	3.61

* RI, retention index as determined on a HP-5MS column using the homologous series of *n*-hydrocarbons.

**Table 2:** Constituents identified from the essential oil of *Artemisia subdigitata* aerial parts.

RI *	Compound	Percent Composition
927	α-Thujene	1.43
939	α-Pinene	0.49
952	Camphene	2.28
977	Sabinene	0.64
981	β-Pinene	7.38
991	β-Myrcene	4.54
1010	δ-3-Carene	0.23
1032	1,8-Cineole	12.26
1038	(*Z*)-β-Ocimene	1.75
1049	2,6-Dimethyl-2,6-octadiene	0.27
1057	γ-Terpinene	2.26
1167	Borneol	6.23
1175	Terpinen-4-ol	3.39
1191	α-Terpineol	1.14
1195	Estragole	4.25
1235	Geraniol	2.82
1297	Acetophenone	1.56
1356	Eugenol	5.87
1374	Copaene	0.29
1420	Caryophyllene	2.84
1436	β-Bergamotene	0.88
1454	α-Caryophyllene	1.15
1457	(*E*)-β-farnesene	1.64
1483	α-Curcumene	10.77
1488	Acenaphthene	3.83
1492	α-Zingiberene	4.62
1505	α-Farnesene	1.64
1512	δ-Amorphene	0.55
1521	δ-Cadinene	1.78
1524	β-Sesquiphellandrene	0.22
1578	Spathulenol	4.09
1584	Caryophyllene oxide	3.14
1608	Humulene oxide II	2.08
	Total	98.31
	Monoterpenoids	51.36
	Sesquiterpenoids	39.52
	Others	7.43

* RI, retention index as determined on a HP-5MS column using the homologous series of *n*-hydrocarbons

**Table 3:** Toxicity of the essential oils of *Artemisia giraldii* and *A. subdigitata* against *Sitophilus zeamais* adults.

Toxicity	Essential oil	LD_50_/LC_50_ *	95% FL	Slope ± SE	Chi square (χ^2^ )
Contact	*A. giraldii*	40.51	37.83–44.89	3.26 ± 0.39	7.13
	*A. subdigitata*	76.34	70.77–82.14	2.37 ± 0.21	9.48
	Pyrethrum extract	4.29 ^a^	3.86–4.72	0.72 ± 0.01	13.51
Fumigant	*A. giraldii*	6.29	5.48–7.38	2.59 ± 0.30	8.12
	*A. subdigitata*	17.01	15.57–18.78	1.67 ± 0.14	15.90
	MeBr	0.67 ^b^	-	-	-

* Contact toxicity: LD_50_ = μg/adult; Fumigant: LC_50_ = mg/L air, ^a^ Liu *et al.* [12]; ^b^ Liu & Ho [28].
